# Cardiac amyloidosis: the need for early diagnosis

**DOI:** 10.1007/s12471-019-1299-1

**Published:** 2019-07-29

**Authors:** M. I. F. J. Oerlemans, K. H. G. Rutten, M. C. Minnema, R. A. P. Raymakers, F. W. Asselbergs, N. de Jonge

**Affiliations:** 1 Department of Cardiology, Division of Heart and Lungs, University Medical Center Utrecht, Utrecht, The Netherlands; 2 Department of Hematology, Cancer Center, University Medical Center Utrecht, Utrecht, The Netherlands; 3 Institute of Cardiovascular Science, Faculty of Population Health Sciences, University College London, London, UK; 4 Institute of Health Informatics, Faculty of Population Health Sciences, University College London, London, UK

**Keywords:** Amyloidosis, Heart, Awareness, Diagnosis, Treatment, Left ventricular hypertrophy, HFpEF

## Abstract

Amyloidosis is a collection of systemic diseases characterised by misfolding of previously soluble precursor proteins that become infiltrative depositions, thereby disrupting normal organ structure and function. In the heart, accumulating amyloid fibrils lead to progressive ventricular wall thickening and stiffness, resulting in diastolic dysfunction gradually progressing to a restrictive cardiomyopathy. The main types of cardiac amyloidosis are amyloid light chain (AL) amyloidosis caused by an underlying plasma cell dyscrasia, amyloid transthyretin (TTR) amyloidosis of wild-type (normal) TTR at older age (ATTRwt) and hereditary or mutant amyloid TTR (ATTRm) in which a genetic mutation leads to an unstable TTR protein. Overall survival is poor once heart failure develops, underlining the need for early referral and diagnosis. Treatment for AL amyloidosis has improved markedly over the last decades, and TTR amyloidosis gene silencers and orally available transthyretin stabilisers are ready to enter the clinical arena after recent positive outcome trials. Novel therapies aiming at fibril degradation with monoclonal antibodies are under investigation. In this review, we focus on ‘red flag’ signs and symptoms, diagnosis and management of cardiac amyloidosis which differs considerably from the general management of heart failure. Only by increasing awareness, prognosis for patients with this devastating disease can be improved.

**Electronic supplementary material:**

The online version of this article (10.1007/s12471-019-1299-1) contains supplementary material, which is available to authorized users.

## Key messages

- The main types of cardiac amyloidosis are light chain amyloidosis and transthyretin amyloidosis.
- Cardiac amyloidosis should be considered in patients with:
  - heart failure with preserved ejection fraction;
  - unexplained left ventricular hypertrophy;
  - heart failure refractory to conventional therapy.
- Early diagnosis and therefore increased awareness is necessary to improve outcome.

## Introduction

**Fig. 1:**
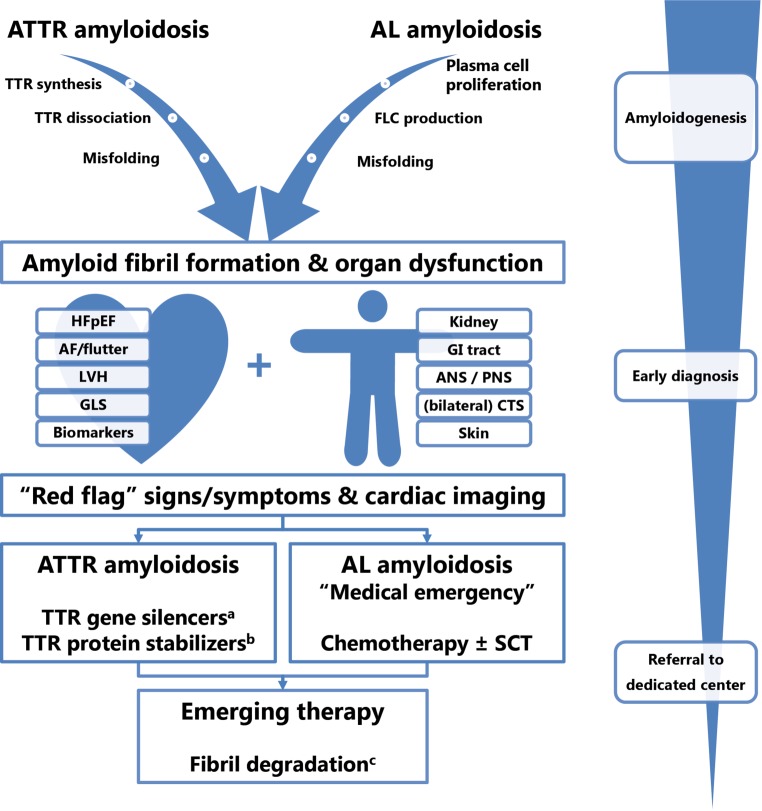
Overview of amyloidogenesis, clues for early diagnosis and treatment of TTR and light chain cardiac amyloidosis. ^a^siRNA (patisiran), antisense oligonucleotide (inotersen). ^b^tafamidis (selective stabiliser); diflunisal (non-selective stabiliser). ^c^antibody-mediated phagocytosis, doxycycline. *ATTR* amyloid transthyretin, *AL* amyloid light chain, *TTR* transthyretin, *FLC* free light chains, *HFpEF* heart failure with preserved ejection fraction, *AF* atrial fibrillation, *LVH* left ventricular hypertrophy, *GLS* global longitudinal strain, *GI* gastro-intestinal, *ANS* autonomous nervous system, *PNS* peripheral nervous system, *CTS* carpal tunnel syndrome, *SCT* stem cell transplantation

Amyloidosis is a collection of systemic diseases characterised by misfolding of previously soluble precursor proteins that become insoluble aggregates disrupting normal organ structure and function [1]. A large number of precursor proteins are able to form organised structures called amyloid fibrils [2, 3]. Although these fibrils mainly infiltrate the heart and kidneys, the peripheral nervous system, gastro-intestinal tract and soft tissues are also affected (Fig. 1). The main types of cardiac amyloidosis are amyloid light chain (AL) amyloidosis caused by an underlying plasma cell dyscrasia, amyloid transthyretin (ATTR) amyloidosis of either non-mutant (wild-type) TTR seen at older age (ATTRwt) and hereditary or mutant TTR (ATTRm) amyloidosis in which a genetic mutation leads to an unstable transthyretin (TTR) protein [4]. Accumulating amyloid depositions lead to ventricular wall thickening and diastolic dysfunction, gradually progressing to a restrictive cardiomyopathy in advanced stage disease. Once diagnosed, the treatment is two-fold: correcting the underlying amyloidogenic process and managing symptoms related to cardiac (and other organ) involvement. Cardiac dysfunction is the major determinant of survival, but diagnosis is often delayed given the aspecific symptoms at onset [5, 6].

In this review, we provide an overview of the main forms of cardiac amyloidosis, its clinical characteristics (Tab. 1), diagnostic approach and management. Several ‘red flags’ are given to aid clinicians in the early recognition (Tab. 2). Finally, we discuss specific considerations on the pharmacological management of cardiac amyloidosis which differs from the general management of heart failure (Tab. 3). Suggested further reading is summarised in Supplementary Table 1.

**Table 1:** Main types of cardiac amyloidosis and clinical characteristics

Type	Precursor Protein	Underlying disorder	Organ involvement	Sex and age (yrs.)	Typical clinical presentation
AL	Immunoglobin light chains	Plasma cell dyscrasias	Kidney Heart Liver ANS, PNS	Either sex, >50	Heart failure with multi-organ involvement including nephrotic syndrome, autonomic dysfunction (orthostatic hypotension, diarrhoea, bladder disorder), peripheral neuropathy, macroglossia and periorbital purpura. Severe hypotension after ACEi/ARB
ATTRwt (‘SSA’)	Wild-type transthyretin	Ageing	Heart	Male > Female >70	Slowly progressive energy decline, exercise intolerance, weight loss and gastro-intestinal complaints. Left and right sided congestive heart failure with normal systolic function and clear diastolic dysfunction, arrythmias. History of CTS
ATTRm	Mutant transthyretin	TTR gene mutation	Nervous system^a^ Heart	Male > Female >40^b^	Slowly progressive heart failure symptoms (as in ATTRwt) with (invalidating) peripheral or autonomic neuropathy. Family history of neurological disease

*AL* amyloid light chain, *ATTRwt* wild-type amyloid transthyretin, *ATTRm* mutant amyloid transthyretin, *SSA* senile systemic amyloidosis, *ANS* autonomic nervous system, *PNS* peripheral nervous system, *ACEi* angiotensin-converting enzyme inhibitor, *ARB* angiotensin receptor blocker, *CTS* carpal tunnel syndrome

^a^Autonomic and peripheral nervous system involvement can lead to similar symptoms as in AL amyloidosis

^b^Mutation dependent, African-Americans with V122I may present at an older age around >60 years

**Table 2:** Red flags for suspicion of cardiac amyloidosis

Diagnostic	Red flag
History taking	Carpal tunnel syndrome (bilateral) in an elderly patient with increased wall thickness on echocardiography, family history of neuropathy or SCD; Complaints of sensory peripheral neuropathy, foamy urine and or bleeding
ECG	Low QRS voltage or disproportionally low voltage in the presence of increased left ventricular wall thickness/LVH
ECG	Pseudo-infarct pattern in the absence of wall motion abnormalities on echocardiography
Echocardiography	Symmetrical LVH (and RVH) in the absence of aortic stenosis or longstanding hypertension
Echocardiography	Preserved LVEF, but reduced GLS with apical sparing (see also Fig. 1)
CMR	Transmural or subendocardial LGE not related to a coronary artery territory, diffuse atrial LGE, RV LGE, suboptimal nulling
CMR	Increased native T1 values, increased extracellular volume, myocardial oedema (T2)
Laboratory testing	Disproportionally high level of NT-proBNP, chronically elevated troponin at low level with normal CAG

*SCD* sudden cardiac death, *ECG* electrocardiogram, *LVH* left ventricular hypertrophy, *RVH* right ventricular hypertrophy, *LVEF* left ventricular ejection fraction, *GLS* global longitudinal strain, *CMR* cardiac magnetic resonance, *LGE* late gadolinium enhancement, *RV* right ventricle,* CAG* coronary angiography

**Table 3:** Pharmacological management in cardiac amyloidosis

Clinical situation	Medication	Comment
Fluid retention, oedema and orthopnoea	Loop diuretic, often combined with mineralocorticoid receptor antagonist (MRA)	Careful titration, avoid underfilling
Supraventricular arrhythmias (atrial fibrillation/flutter)	β-blocker	Only in case of very high heart rate; generally to be avoided due to rate-dependent maintenance of cardiac output in advanced stage
	Amiodaron	Generally well tolerated. Capable of keeping sinus rhythm
	Non-dihydropyridine calcium channel blockers (i.e. verapamil, diltiazem)	Contra-indicated as toxicity may occur quickly due to abnormal binding to amyloid fibrils; Same effect as with β‑blocker
	Digoxin	Contra-indicated as toxicity may occur quickly due to abnormal binding to amyloid fibrils
	Anticoagulant therapy	Should be considered even in sinus rhythm or low CHA2DS2VASC score due to a high risk of atrial thrombus in case of atrial dysfunction
QT-prolonging medication	Especially antipsychotics (haloperidol, quetiapine, olanzapine), tricyclic antidepressants (amitriptyline, nortriptyline, citalopram), anti-emetics (metoclopramide, ondansetron), antibiotics (ciproxin, azoles)^a^	Should be used with careful monitoring of QRS duration

^a^For a complete list of QT-prolonging mediation, see www.QTdrugs.org

## The Need For Early Diagnosis

The clinical presentation is variable and education of physicians is needed to ensure that amyloidosis is considered as diagnosis [2, 5]. Early diagnosis is crucial for several reasons: a) overall survival is poor once cardiac involvement is present [7, 8]; b) chemotherapy followed by stem cell transplantation has improved prognosis for AL amyloidosis significantly [5, 8]; c) antibody-mediated fibril phagocytosis as well as TTR gene silencers and protein stabilisers are emerging including recent positive outcome trials on all-cause mortality and hospitalisation [9, 10]. Thus, increased awareness is necessary to improve patient outcome with this lethal disease.

## Epidemiology

Cardiac amyloidosis was previously regarded a rare condition. AL cardiac amyloidosis (AL-CA) is the most prevalent type responsible for ±70% of all newly diagnosed patients with cardiac amyloidosis [2, 11]. A limited number of epidemiologic studies have been reported, but the incidence of AL amyloidosis in the Western world is 8–12 per million persons per year [5, 11]. Data on ATTR amyloidosis are even more sparse; a recent Swedish study reported an incidence of 2.0 per million inhabitants per year [12]. However, it is commonly overlooked due to the more gradual course and aspecific symptoms in the elderly. Underlying ATTR cardiac amyloidosis (ATTR-CA) is frequently present in patients with aortic stenosis (~10%) and heart failure with preserved ejection fraction (HFpEF) aged 75 or older, although the clinical relevance of amyloid depositions reported on autopsy remains unknown [13–15]. Using bone scintigraphy ATTR-CA was detected in 13% of patients with HFpEF and in 10% of patients who recently underwent carpal tunnel surgery [16, 17]. Prevalence of cardiac amyloidosis is increasing due to better imaging modalities and the aging population [5, 11].

## Clinical Characteristics

### Cardiac symptoms

Patients with cardiac amyloidosis develop diastolic dysfunction leading to HFpEF complaining of fatigue, shortness of breath and oedema [2, 8, 18]. Syncope on exertion may occur due to the ‘fixed’ and relatively low stroke volume next to conduction disorders (bundle branch block and/or atrioventricular block) after infiltration of the conduction system. Depositions in the small (coronary) vessels, may lead to angina and jaw or leg claudication. Atrial dysfunction and atrial fibrillation due to atrial amyloid depositions may cause thromboembolism requiring antithrombotic therapy. Heart failure symptoms, left ventricular hypertrophy on imaging and signs and symptoms of other organ involvement offer an important clue, as summarised in Tab. 2.

### AL amyloidosis

AL amyloidosis is a primary haematological multi-organ disease, although heart and kidneys (70–80%) are predominately affected, presenting at the age of 55–60 [7, 8, 19]. Especially renal involvement leading to nephrotic syndrome may be the first manifestation next to peripheral neuropathy, autonomous dysfunction and gastro-intestinal complaints (Tab. 1; [1, 2]). Periorbital bruising and macroglossia are considered pathognomic but relatively rare. An important clue for cardiologists is severe hypotension after initiation of angiotensin-converting enzyme (ACE) inhibitors or angiotensin receptor blocker (ARB) therapy or clinical deterioration after introduction of β‑blockade. Isolated cardiac amyloidosis is found in approximately 5%. Carpal tunnel syndrome is less common then in ATTR amyloidosis. Multiple myeloma can be identified in 10–15%, but most patients have <20% plasma cells with bone marrow biopsy. Severe cardiac involvement at diagnosis often prevents patients from getting optimal therapy leading to a median survival of 6–12 months, which is worse than in ATTR-CA and probably caused by direct cardiotoxic effects of light chains [6, 20]. Moderate or little cardiac involvement has a much better outcome.

### ATTR amyloidosis

In ATTRwt amyloidosis, the misfolding of TTR is associated with age (formerly called senile systemic amyloidosis, SSA), mainly affecting the heart, although it has a systemic distribution [21]. Depositions in other organs are generally well tolerated, mean age at presentation is >70 years predominantly in males complaining of heart failure or atrial arrhythmias and conduction disorders. Asymmetrical left ventricular hypertrophy and ejection fraction <50% do occur and women are affected in 20% of cases [22]. As shown in Tab. 2, carpal tunnel syndrome is an important clue [1, 18]. Median survival is about 5 years from diagnosis. In ATTRm amyloidosis, the rate of cardiac versus neurological involvement depends on the underlying TTR mutation of which over 100 have been identified [23]. The most common mutations are the valine-to-isoleucine mutation at position 122 (Val122I), carried by 3–4% of the African-Americans leading to late-onset cardiac amyloidosis and the Val30Met mutation with a high prevalence in northern Portugal and Sweden [24]. The Thr60Ala (Ireland), Leu111Met (Denmark) and Ile68Leu (Italy) mutations have a more malignant course with onset at the age of 40 [5, 21]. The clinical presentation is comparable to ATTRwt amyloidosis with the addition of peripheral or autonomic nervous system involvement (Tab. 2); overall survival is estimated at 5–8 years [23].

## Diagnosis Of Cardiac Amyloidosis

‘Red flag’ signs and symptoms contribute to early recognition (Tab. 2) and suspicion should increase in patients with unexplained HFpEF and/or left ventricular hypertrophy [2, 5, 8]. Data on diagnostic performance are mostly lacking. The reader is referred to a pooled cohort covering clinical findings, a recent meta-analysis on bone scintigraphy and the Supplementary Table 1 [17, 22].

In Fig. 4, a diagnostic flow chart is provided to establish either AL-CA or ATTR-CA. The differential diagnosis consists of the most common genetic and non-genetic disorders causing a hypertrophic cardiomyopathy, next to the easily excluded long-standing hypertension and aortic stenosis. Importantly, aortic stenosis and clonal immunoglobulin abnormalities may coexist with ATTR-CA [5, 7, 22]. For adequate typing, the combination of bone scintigraphy and testing for monoclonal proteins is crucial [2, 5]. Especially in AL amyloidosis, timely diagnosis is essential for therapeutic outcome.

### Electrocardiography

The typical ECG in cardiac amyloidosis is characterised by low QRS voltage due to amyloid infiltration, seen in approximately 50–60% in AL-CA but in only 20% in ATTR-CA [6, 8]. Therefore, an important clue is a discrepancy between the left ventricular wall thickness and QRS voltage (*red flag*, Tab. 2). In approximately 50–70% of patients pseudo-infarct patterns are present, eventually leading to unnecessary coronary angiography (*red flag*, Tab. 2; [22]). The *P* wave may reflect prolonged atrial conduction or atrial dilatation. Conduction disorders occur frequently, the presence of atrioventricular block in patients with left ventricular hypertrophy should raise suspicion.

### Echocardiography

**Fig. 2:**
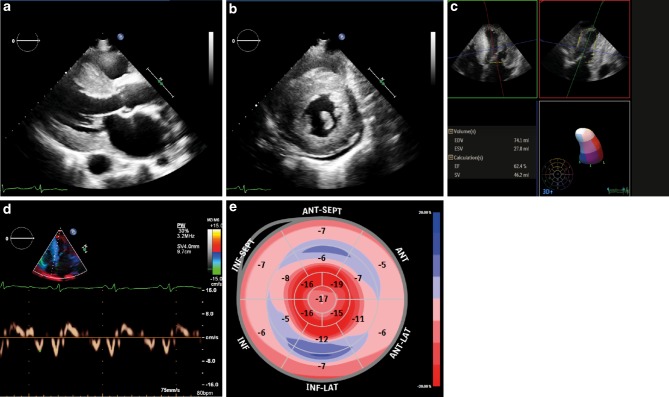
Echocardiography in cardiac amyloidosis. **a**–**b** Granular echogenic appearance of the left ventricular wall with clear hypertrophy and some pericardial effusion. Left ventricular ejection fraction is preserved (**c**), septal tissue Doppler longitudinal movement is reduced (**d**). Longitudinal strain analysis from the 3 apical views showing characteristic apical sparing (bull’s eye) with reduced strain at the mid and basal level (**e**)

Echocardiographic findings in cardiac amyloidosis are a classic example of an infiltrative cardiomyopathy. Increased echogenicity (i.e. granular or ‘speckled’ myocardium) had good sensitivity and specificity using fundamental imaging before harmonic imaging was introduced and is not reliable anymore [5, 25]. Major findings are increased left ventricular wall thickness (>12 mm, either symmetrical or asymmetrical) in combination with the right ventricular free wall, thickened valves and pericardial effusion (Fig. 2). Global longitudinal left ventricular strain analysis may reveal a pattern of prognostically important apical ‘sparing’, but the specificity for cardiac amyloidosis needs further study. Left ventricular ejection fraction (LVEF) is mostly preserved (*red flag*, Tab. 2; [25]). Reduced tissue Doppler velocities of the mitral annulus are common, diastolic dysfunction is frequently present including a restrictive physiology: high E/A ratio, even absence of the A wave, shortened deceleration time, high E/e’ and severely increased atrial volumes. Importantly, atrial dysfunction can also be demonstrated by an abnormal left atrial strain and may lead to the formation of thrombi even in the absence of atrial fibrillation (atrial standstill) [26].

### Cardiac magnetic resonance (CMR)

**Fig. 3:**
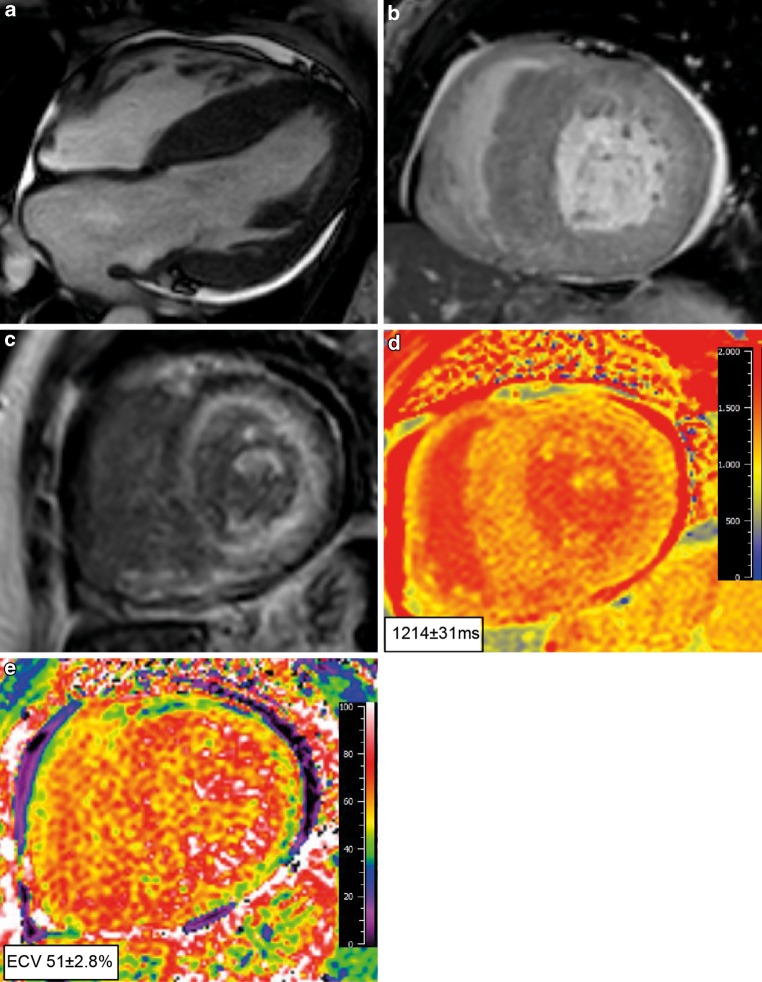
Cardiac magnetic resonance imaging in cardiac amyloidosis. **a**–**b** Cardiac magnetic resonance imaging showing a 4‐chamber and short axis view with left ventricular hypertrophy mainly in the septal region. Corresponding short axis view showing generalised subendocardial late gadolinium enhancement not fitting a coronary territory. Note the reduced signal of the blood pool (dark blood), which is specific for cardiac amyloidosis (**c**). Native T1 mapping analysis showing an increased T1 value of 1214 ± 31 ms (**d**) and subsequent ECV map which is increased giving a value of 51 ± 2.8% (**e**), both acquired with a field strength of 1.5 T

The advantage of CMR, besides a higher resolution, is the possibility to further characterise the myocardium using late gadolinium enhancement (LGE) imaging and T1 mapping, a quantitative technique able to detect diffuse myocardial abnormalities including amyloid burden [27]. Transmural or subendocardial LGE not fitting a coronary artery territory or including the right ventricle may be the first clue, next to suboptimal nulling due to altered gadolinium kinetics (Fig. 3). Native T1 mapping (before contrast) is typically prolonged. Postcontrast T1 mapping is decreased as the myocardial tissue reaches the null crossing at an earlier or similar inversion time as the blood pool, subsequently resulting in an increased extracellular volume (ECV) generally >40% (*red flag*, Tab. 2). In both ATTR-CA and AL-CA, T2 values indicative of myocardial oedema are increased and T2 is a predictor of prognosis in AL-CA [28]. CMR, similar to echocardiography, cannot differentiate between amyloidosis types.

### Nuclear imaging

Phosphate-based technetium tracers (^99^mTc-PYP, ^99^mTc-MDP, ^99^mTc-DPD), normally used for bone scintigraphy, accumulate in the presence of ATTR-CA although the exact mechanism is unclear. Uptake in AL-CA, Fabry disease and hypertrophic cardiomyopathy is absent or low [17]. Visual grading is based on the work by Perugini ranging from grade 0 (no uptake) to grade 3 if cardiac uptake is higher than in bone [29]. Grade 2 and 3 uptake in the absence of a monoclonal protein showed a 100% specificity and positive predictive value for ATTR-CA without the need for additional histology [30]. Importantly, in all other cases (i.e. low-grade uptake, positive light chains) cardiac and/or tissue biopsy is necessary to rule out AL-CA and more rare forms of amyloidosis (AA, AApoA1) [3]. Although positive emission tomography (PET) using ^18^F-Florbetapir and ^11^C-PiB to image β‑amyloid looks promising, it is not routinely used yet [31, 32].

### Endomyocardial biopsy (EMB)

**Fig. 4:**
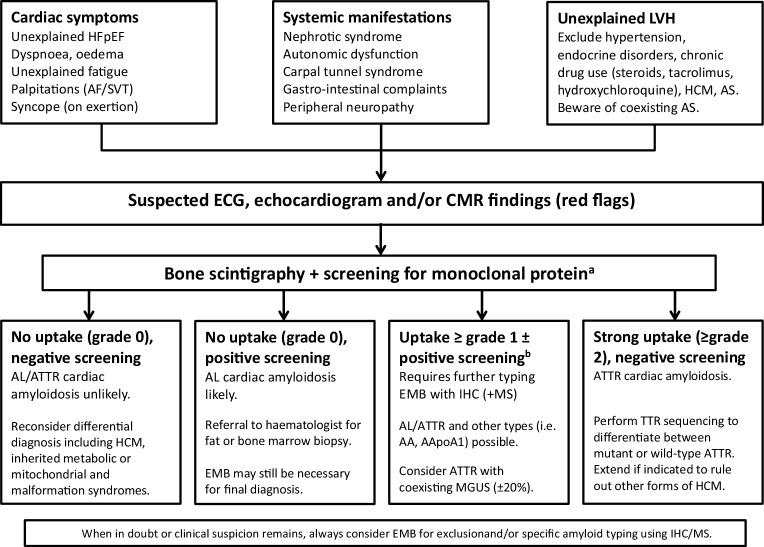
Flow chart for the diagnosis of cardiac amyloidosis. ^a^serum-free light chain assay, serum/urine immunofixation. ^b^Perugini grade 1 or 2 with or w/o positive AL amyloidosis screening, grade 3 with positive AL amyloidosis screening. *HFpEF* heart failure with preserved ejection fraction,* AF* atrial fibrillation, *SVT* supraventricular tachycardia, *LVH* left ventricular hypertrophy, *HCM* hypertrophic cardiomyopathy, *AS* aortic stenosis, *ECG* electrocardiogram, *CMR* cardiac magnetic resonance, *AL* amyloid light chain, *ATTR* amyloid transthyretin, *EMB* endomyocardial biopsy, *IHC* immunohistochemistry, *MS* mass spectrometry, *AA* amyloid serum A protein, *AApoA1* amyloid apolipoprotein A1, *MGUS* monoclonal gammopathy of unknown significance, *TTR* transthyretin

In case of ongoing clinical suspicion, nuclear imaging with low-grade or high-grade tracer uptake in combination with positive monoclonal protein, EMB is necessary to correctly type the underlying amyloidosis (Fig. 4). EMB should be performed by experienced clinicians and trained pathologists. Congo red will demonstrate amyloid depositions as red staining under bright light and shows apple-green birefringences under polarised light, which should be followed by immunohistochemistry. Mass spectrometry after laser dissection is increasingly being used to determine the specific amyloid type especially when there is also some degree of monoclonal gammopathy [3, 7].

### Laboratory testing, biomarkers, tissue biopsy and genetic testing

Standard laboratory screening may reveal kidney and liver involvement. Serum/urine immunofixation in combination with a serum-free light chain assay will evaluate light chain abnormalities [1, 8]. In chronic kidney disease impaired renal filtration leads to altered κ/λ ratios [5]. N‑terminal prohormone brain natriuretic peptide (NT-proBNP) levels are often elevated to a disproportionally high levels due to toxicity of the precursor proteins (*red flag*, Tab. 2). Troponins are chronically elevated without rise/fall. Both troponin and NT-proBNP have prognostic implications in AL-CA and in ATTR-CA [20, 33]. Importantly, diagnosing AL-CA based on plasma dyscrasias and a positive tissue biopsy alone can be insufficient since 20% of patients with ATTR-CA also have a non-related clonal immunoglobulin abnormality [7]. When AL amyloidosis is suspected, final diagnosis is established in close collaboration with the haematologists for which a bone marrow biopsy is essential to confirm plasma cell dyscrasia. If a fat biopsy is performed, a negative result does not rule out amyloidosis because of lower sensitivity [23]. In case of ATTR-CA, TTR gene sequencing is recommended to confirm or exclude ATTRm, as well as other forms of hypertrophic cardiomyopathy, if indicated.

## Therapy

After diagnosis, staging is necessary to assess treatment eligibility and prognosis [34]. This is based on the Mayo Clinic system for AL amyloidosis using troponin, NT-proBNP and serum-free light chain and performance scale [20, 35]. The revised European staging also includes a subgroup of stage IIIb (NT-proBNP >8500 pg/ml) and a dismal prognosis. For ATTR amyloidosis, estimated glomerular filtration rate (eGFR) reflecting renal dysfunction was suggested to improve stratification [21, 33]. Therapeutic goals are the management of heart failure and interfering with the underlying amyloidogenic process [5, 6].

### Cardiac supportive therapy

Pharmacological management in cardiac amyloidosis is different from the general management of heart failure as commonly used medication can have important negative consequences (Tab. 3). Diuretics are the cornerstone in symptom management often combined with mineralocorticoid receptor antagonists. This is more challenging in AL-CA with multiple organs affected, giving an increased risk for renal failure or orthostatic hypotension. To avoid underfilling, sodium and fluid restriction are important to keep patients euvolemic with the smallest amount of diuretics possible [8]. Midodrine is sometimes used to treat orthostatic hypotension, ACE inhibitors and ARB are poorly tolerated especially in AL-CA [7]. The use of β‑blockers should generally be avoided; an increased heart rate is the only way to maintain adequate cardiac output in advanced disease. Non-dihydropyridine calcium channel blockers (i.e. verapamil, diltiazem) and digoxin are contra-indicated as toxicity may occur quickly due to abnormal binding to amyloid fibrils [36, 37]. In case of supraventricular arrhythmias, amiodarone should be considered next to catheter ablation. Importantly, anticoagulation should be considered even in patients with sinus rhythm or low CHA_2_DS_2_VASC score (congestive heart failure, hypertension, age ≥75 years [doubled], diabetes mellitus, prior stroke [doubled]-vascular disease, age 65–74, sex category) given the high incidence of atrial thrombi (atrial standstill), although randomised trials are lacking [26]. Careful monitoring for thrombocytopenia is necessary after chemotherapy in AL-CA. Ventricular arrhythmias do occur, but implantable cardioverter defibrillator (ICD) therapy is usually not indicated as sudden cardiac death (SCD) is mostly caused by bradycardia or adequate rhythm with pulseless electrical activity [38, 39]. According to the general guidelines for bradypacing, implantation of a pacemaker should be considered, but it remains unclear whether this prevents SCD. QT-prolonging medication should be given under careful monitoring, as especially anti-emetics or QT-prolonging prophylactic antibiotics necessary during chemotherapy can be challenging. Finally, only in highly selected patients a heart transplantation may be considered in isolated AL-CA in combination with chemotherapy and stem cell transplantation. Widespread experience is lacking and this has not been performed in the Netherlands [40].

### Treatment of AL amyloidosis

Patients must be referred to the haematologist to initiate chemotherapy targeting the clonal plasma cells [7, 34]. Treatment-related mortality is high as the treatment by itself can be cardiotoxic leading to worsening heart failure. Although chemotherapy followed by autologous stem cell transplantation (SCT) is the preferred therapy to obtain complete remission, only a minority of patients are eligible after patient selection [7, 8, 41]. Severe cardiac involvement is an important contraindication (serum NTproBNP level is >5000 pg/ml, serum troponin T level is >0.06 ng/ml, LVEF <45%, New York Heart Association [NYHA] class ≥ III) next to severe kidney dysfunction, hypotension, age >70 years and impaired performance status [34, 35].

### Treatment of ATTR amyloidosis

Although classically seen as a lethal disease without specific treatment, several disease-modifying drugs have been developed recently interfering at different points in the TTR amyloidogenesis pathway [5]. Gene silencers interfere with TTR protein synthesis in the liver including patisiran, a small interfering ribonucleicacid (siRNA) in a lipid nanoparticle targeting hepatocytes. Patisiran reduced TTR protein levels in ATTRm patients with neuropathy slowing disease progression (Apollo phase III trial) and led to a reduction in hospitalisation and/or all-cause mortality in the ATTR-CA[10]. Inotersen, an antisense oligonucleotide, showed substantial benefit in ATTRm patients with polyneuropathy but safety concerns were raised as thrombocytopenia and glomerulonephritis were reported [42]. Next to gene silencing, stabilisers preventing TTR dissociation have been investigated. Diflunisal, an old non-steroidal anti-inflammatory drug (NSAID) stabilising the TTR tetramer in patients with ATTRm and polyneuropathy showed a reduction in the rate of progression [43]. It was reasonably tolerated in a small open-labelled study, but larger trials assessing common NSAID side effects (gastro-intestinal bleeding, kidney dysfunction, worsening heart failure) are necessary [44]. Recently, a multicentre placebo-controlled phase III study showed a reduction of all-cause mortality and hospitalisations in both ATTRm and ATTRwt patients treated with the oral TTR stabiliser tafamidis [9]. Other potential options are Green Tea extract, capable of stabilising the TTR protein, and doxycycline combined with biliary acid, lowering TTR depositions. However, these compounds have only been studied in small animal models and an open label study with 20 patients [45]. Liver transplantation in ATTRm is an option in selected cases without cardiac involvement, but timing of this major operation is difficult and follow-up showed an accelerated occurrence of wild-type ATTR after transplantation with extensive cardiac depositions [11].

### Emerging therapies for amyloid fibril degradation

Several trials are underway investigating the application of antibodies recognising amyloid fibrils to induce phagocytosis including an anti-amyloid antibody 11-1F4 (phase I, NCT02245867) and serum amyloid P (SAP) antibody (phase I, NCT01777243) although some safety issues remain to be investigated [5, 46]. Investigation of NEOD001 (phase III, NCT02312206), an antibody targeting circulating and deposited amyloid, was discontinued after a negative futility analysis [47]. Another option is doxycycline showing an inhibitory effect on fibrillogenesis in transgenic AL amyloidosis mice as well as in stage III AL-CA patients in a small retrospective study leading to increased survival [48]. A prospective trial investigating doxycycline is currently enrolling patients [49].

## Conclusions and Outlook

Major advancements have been made in the past decade in the treatment of cardiac amyloidosis. Especially AL-CA is a medical emergency and new chemotherapeutic regimens followed by stem cell transplantation have improved prognosis considerably. For ATTR-CA, TTR gene silencers and stabilisers are awaiting approval by the National Health Care Institute (*Zorginstituut Nederland*), giving patients high expectations. Monoclonal antibodies facilitating breakdown of existing depositions would provide a major breakthrough, making this a reversible disease. Patients will benefit the most if cardiac amyloidosis is diagnosed early, requiring timely referral and work-up in an experienced centre using a multidisciplinary approach. The accompanying costs ask for future cost/benefit analyses (i.e. close monitoring in a national registry) to provide more information on who benefits the most.

Early diagnosis is important given the emerging therapies. If we can increase awareness, the landscape for patients with cardiac amyloidosis will definitely change.

## Caption Electronic Supplementary Material

Supplementary Table 1—Suggested in-depth reviews on clinical manifestation, diagnosis, imaging and prognostic staging
