# Supplementary material for: Cardiac amyloidosis: the need for early diagnosis
Source: Neth Heart J. 2019 Jul 29;27(11):525–36. doi: 10.1007/s12471-019-1299-1 (PMC6823341; doi:10.1007/s12471-019-1299-1)
Supplement: Supplementary file 1 — Supplementary Table 1—Suggested in-depth reviews on clinical manifestation, diagnosis, imaging and prognostic staging [file 12471_2019_1299_MOESM1_ESM.docx]

| **Supplementary Table 1 – Suggested further reading** | | | |
| --- | --- | --- | --- |
| **Reference** | **Brief description** | **Author** | **Year** |
| [1] | Excellent overview of amyloidosis including the systemic manifestations | Dubrey | 2011 |
| [2] | State-of-the-art review on AL cardiac amyloidosis | Falk | 2016 |
| [3] | Recently updated review on AL cardiac amyloidosis | Merlini | 2018 |
| [4] | Excellent review on ATTR amyloidosis | Gertz | 2015 |
| [5] | Important consensus document of the International Society of Amyloidosis on nomenclature and classification | Sipe | 2016 |
| [6] | Important paper describing the (non-biopsy) diagnostic work-up in ATTR cardiac amyloidosis | Gillmore | 2016 |
| [7] | Excellent overview on the diagnostic accuracy of bone scintigraphy in ATTR cardiac amyloidosis | Treglia | 2018 |
| [8] | An overview of key clinical characteristics in ATTR amyloidosis including symptoms, ECG findings and echocardiography in a large multicenter pooled analysis | Gonzalez-Lopez | 2017 |
| [9] | Comprehensive overview of imaging in amyloidosis | Falk | 2014 |
| [10] | Recent paper on CMR in amyloidosis including T1 mapping and quantification of extracellular volume (ECV) | Marinez-Naharro | 2017 |
| [11] | Revised prognostic staging system for AL amyloidosis | Wechalekar | 2013 |
| [12] | Prognostic staging system for ATTR amyloidosis | Grogan | 2016 |

*AL* amyloid light chain, *ATTR* amyloid transthyretin, *CMR* cardiac magnetic resonance

1. Dubrey SW, Hawkins PN, Falk RH. Amyloid diseases of the heart: Assessment, diagnosis, and referral. Heart. 2011;97:75–84.

2. Falk RH, Alexander KM, Liao R, et al. AL (Light-Chain) Cardiac Amyloidosis: A Review of Diagnosis and Therapy. J Am Coll Cardiol. 2016;68:1323–41.

3. Merlini G, Dispenzieri A, Sanchorawala V, et al. Systemic immunoglobulin light chain amyloidosis. Nat Rev Dis Prim. 2018;4:38.

4. Gertz MA, Benson MD, Dyck PJ, et al. Diagnosis, Prognosis, and Therapy of Transthyretin Amyloidosis. J Am Coll Cardiol. 2015;66:2451–66.

5. Sipe JD, Benson MD, Buxbaum JN, et al. Amyloid fibril proteins and amyloidosis: chemical identification and clinical classification International Society of Amyloidosis 2016 Nomenclature Guidelines. Amyloid. 2016;23:209–13.

6. Gillmore JD, Maurer MS, Falk RH, et al. Nonbiopsy Diagnosis of Cardiac Transthyretin Amyloidosis. Circulation. 2016;133:2404–12.

7. Treglia G, Glaudemans AWJM, Bertagna F, et al. Diagnostic accuracy of bone scintigraphy in the assessment of cardiac transthyretin-related amyloidosis: a bivariate meta-analysis. Eur J Nucl Med Mol Imaging. 2018;45:1945–55.

8. González-López E, Gagliardi C, Dominguez F, et al. Clinical characteristics of wild-type transthyretin cardiac amyloidosis: disproving myths. Eur Heart J. 2017;38:1895–904.

9. Falk RH, Quarta CC, Dorbala S. How to image cardiac amyloidosis. Circ Cardiovasc Imaging. 2014;7:552–62.

10. Martinez-Naharro A, Treibel TA, Abdel-Gadir A, et al. Magnetic Resonance in Transthyretin Cardiac Amyloidosis. J Am Coll Cardiol. 2017;70:466–77.

11. Wechalekar AD, Schonland SO, Kastritis E, et al. A European collaborative study of treatment outcomes in 346 patients with cardiac stage III AL amyloidosis. Blood. 2013;121:3420–7.

12. Grogan M, Scott CG, Kyle RA, et al. Natural History of Wild-Type Transthyretin Cardiac Amyloidosis and Risk Stratification Using a Novel Staging System. J Am Coll Cardiol. 2016;68:1014–20.
